# Application of Raman Spectroscopic Methods in Food Safety: A Review

**DOI:** 10.3390/bios11060187

**Published:** 2021-06-08

**Authors:** Marlen Petersen, Zhilong Yu, Xiaonan Lu

**Affiliations:** 1 Food, Nutrition and Health Program, Faculty of Land and Food Systems, The University of British Columbia, Vancouver, BC V6T 1Z4, Canada; marlenp@mail.ubc.ca (M.P.); zhilong.yu@mail.mcgill.ca (Z.Y.); 2 Department of Food Science and Agricultural Chemistry, Faculty of Agricultural and Environmental Sciences, McGill University, Saint-Anne-de-Bellevue, QC H9X 3V9, Canada

**Keywords:** SERS, Raman imaging, micro-Raman spectroscopy, food hazards, rapid detection

## Abstract

Food detection technologies play a vital role in ensuring food safety in the supply chains. Conventional food detection methods for biological, chemical, and physical contaminants are labor-intensive, expensive, time-consuming, and often alter the food samples. These limitations drive the need of the food industry for developing more practical food detection tools that can detect contaminants of all three classes. Raman spectroscopy can offer widespread food safety assessment in a non-destructive, ease-to-operate, sensitive, and rapid manner. Recent advances of Raman spectroscopic methods further improve the detection capabilities of food contaminants, which largely boosts its applications in food safety. In this review, we introduce the basic principles of Raman spectroscopy, surface-enhanced Raman spectroscopy (SERS), and micro-Raman spectroscopy and imaging; summarize the recent progress to detect biological, chemical, and physical hazards in foods; and discuss the limitations and future perspectives of Raman spectroscopic methods for food safety surveillance. This review is aimed to emphasize potential opportunities for applying Raman spectroscopic methods as a promising technique for food safety detection.

## 1. Introduction

Food safety has become an important issue as our food system is vulnerable. Contaminated food has been shown to cause severe health problems and death as well as impact economic growth [1]. According to the World Health Organization, unsafe food causes 600 million illnesses and 42,000 death each year worldwide [2]. Foods can be contaminated unintentionally or intentionally in the food supply chain by three classes of contaminants: (1) microbes (e.g., bacteria, virus, and fungi), (2) chemicals (e.g., toxins, pesticides, adulterants, and allergens), and (3) physical contaminants (e.g., metals, glass, plastic, human hairs, and rocks) [3]. As large physical contaminants can easily be detected by visual inspection, sieves, or screening machines, we focus on the determination of plastic particles as they are widely found in packaging materials.

Early detection of contaminants in the food supply chain is critical to ensure safe food. Raman spectroscopy is one of the few techniques that can detect contaminants of all three classes in a rapid, sensitive, non-destructive, and relatively inexpensive manner [3,4]. Compared to other spectroscopy-based methods such as fluorescence and infrared spectroscopy, Raman spectroscopy allows for multiplex detection of different analytes due to their higher spectral resolution and narrower bandwidths. Furthermore, quantitative analysis of analytes can be achieved based on the proportional relationship between Raman signal intensity and analyte concentration [5].

In this review, we summarize the recent development of Raman spectroscopic methods to determine biological, chemical, and physical contaminants found in the food chain, show current challenges, and outline the broad application potential of Raman spectroscopy for food safety.

## 2. Raman Spectroscopic Methods

**Figure 1:**
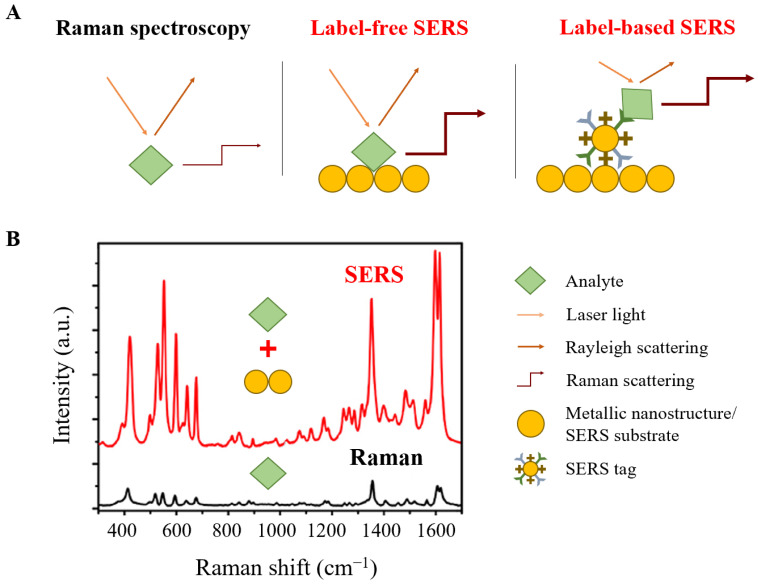
Comparison of Raman spectroscopy and SERS. (**A**) Schematic illustration of Raman spectroscopy, label-free, and label-based SERS. (**B**) Raman (black) and SERS (red) spectrum of pyocyanin. SERS is based on the application of plasmonic gold nanoparticles. Graph visualizes intensity differences between Raman and SERS signals of pyocyanin [5].

Raman spectroscopy is based on an inelastic scattered light phenomenon to detect an analyte via molecular bond vibrations. When a sample is exposed to laser light, a small number of photons are scattered. Most of the scattering is elastically scattered light (i.e., Rayleigh scattering) that has the same frequency as the incident light. Approximately 1 out of 10^6^–10^8^ photons are inelastically scattered (i.e., Raman scattering), resulting in frequency changes (i.e., Raman shifts) between incident and scattered photons as energy is transferred between photon and molecule (Figure 1A). Here, either incident light photon energy is gained by transferring energy from molecules to photons (anti-Stokes Raman scattering), or incident light photon energy is lost by transferring energy from photons to molecules (Stokes Raman scattering) [4,6,7]. Raman spectra are obtained based on the detected Raman shifts (Figure 1B). Each Raman peak in the spectrum is characteristic of a specific molecular bond, which allows for molecular identification of an analyte by generating a specific vibrational fingerprint [5]. The Raman spectroscopic techniques commonly used for food safety detection include micro-Raman spectroscopy, Raman imaging, and surface-enhanced Raman spectroscopy (SERS).

### 2.1. Raman Microscopy and Micro-Raman Spectroscopy

**Figure 2:**
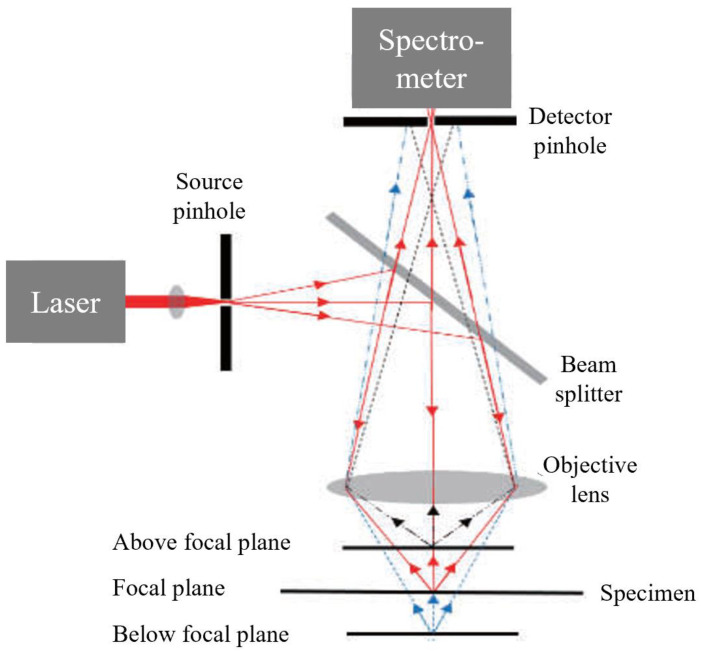
Schematic setup of a confocal Raman microscope [9].

Raman spectroscopy combined with an optical microscope is termed Raman microscopy. In a Raman microscopy setup, Raman scattering is achieved by directing the laser light to the sample via an objective lens [4]. In comparison, the setup of a confocal Raman microscope involves the application of pinhole apertures and is more complex (Figure 2). In confocal micro-Raman spectroscopy, a point light source is directed by a source pinhole and a splitter beam and then focused by an objective lens to a diffraction-limited spot of the specimen. Scattered or emitted light from this spot is collected and collimated by the same objective lens and then passed through a small detector pinhole onto a spectrometer. The detector pinhole acts as a depth selector as it rejects light from out-of-focus sections (e.g., above or below the focal plane) and only collects the signal from the focal point. Confocal microscopes have become a more valuable analytical tool over the past years as it has a better depth resolution and higher image contrast due to stray light suppression [8,9].

### 2.2. Raman Imaging

**Figure 3:**
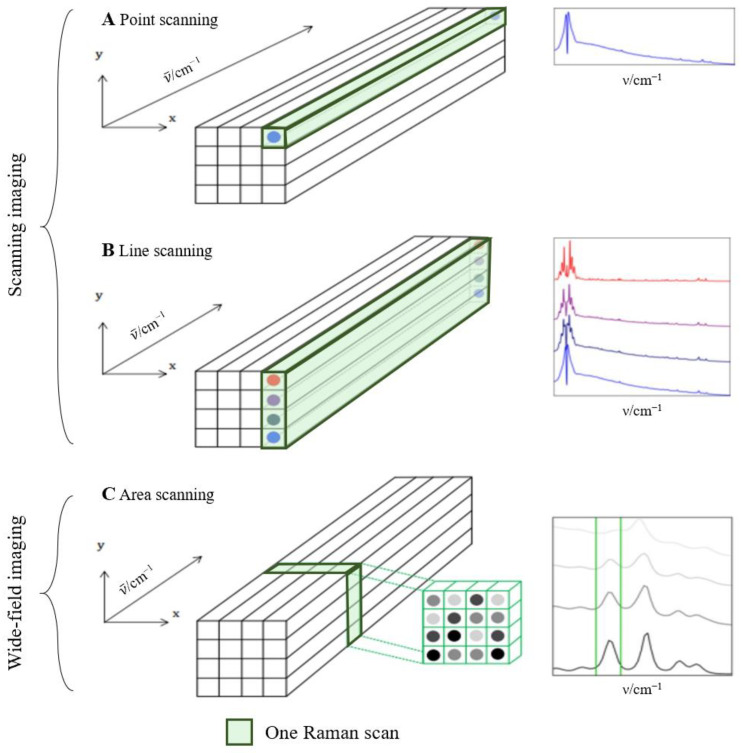
Examples of scanning and wide-field Raman imaging techniques. Raman spectra collection of a 2D image is visualized for (**A**) point scanning, (**B**) line scanning, and (**C**) area scanning [4].

The purpose of Raman imaging is to visualize the distribution of components by chemical properties in a sample [4]. Raman imaging combines spatial information (i.e., x and y spatial dimensions) with Raman spectroscopy (i.e., wavelength dimension) (Figure 3). Thus, each pixel in the image corresponds to a Raman spectrum that is compared to an established Raman database to determine a specific analyte or spectral background measurements in this location. Raman imaging mostly does not require sample preparation [4,10].

There are two approaches to perform Raman imaging, namely scanning imaging and wide-field imaging. Scanning imaging commonly uses a confocal microscope and can be performed in two ways. (1) Point scanning collects a Raman spectrum at each spatial location one by one (Figure 3A). The sample is moved from one specific point to the next on a high-precision stage that controls the lateral and axial coordinates of the sample. Advantages of this method include high spectral resolution and full spectral coverage. However, it is time-consuming and causes laser-induced sample damage. (2) Compared to point scanning, line scanning widens the spatial range of each scan by applying a laser line and thus acquires a line of spatial and spectral information for each measurement (Figure 3B). The sample is moved on an automated stage perpendicular to the incident laser line. Although line scanning results in a weaker Raman scattering signal compared to point scanning due to a lower laser power per area, it still has a high spectral resolution and is faster than point scanning. In wide-field Raman imaging, an entire sample area is illuminated with laser light, and its spatial information is obtained in one scan without relative movement between the laser and the sample. Area scanning is one approach of wide-field imaging and is based on selecting a spectral slice of Raman scattering that permits the specific wavenumber range (Figure 3C). Although this method is rapid, a major limitation is the difficult discrimination of spectral data [4,10].

### 2.3. SERS

The major limitation of conventional Raman spectroscopy is the very weak Raman scattering signal. As only a very small fraction of the incident photons is scattered inelastically (approximately 1 out of 10^6^–10^8^ photons), Raman spectroscopy may fail to detect low concentrations of molecules, limiting its application in the food industry [5,6,7]. SERS is a Raman spectroscopic technique that can overcome inherently weak Raman scattering signal by using SERS substrates (e.g., noble metal nanoparticles or rough metal surfaces). These metallic nanostructures can enhance the low-concentration single-molecule Raman signal by several orders of magnitude, typically ranging between 10^7^ and 10^14^ (Figure 1B) [5,6,11].

Current SERS methods are based on two strategies, termed label-free (direct) and label-based (indirect) SERS (Figure 1A). The label-free SERS method relies on the mutual interaction of an analyte with the SERS substrate and directly detects its intrinsic fingerprint. Compared to label-based SERS methods, this method has several advantages including simplicity, high speed, lower cost, and no interference with other components as no SERS tags are required. Label-based SERS methods use SERS tags that comprise specific Raman reporting molecules for binding to the SERS substrate and target recognition elements for capturing analytes. Advantages of label-based over label-free SERS methods include multiplex detection, higher sensitivity, and repeatability [11,12].

### 2.4. Raman Spectral Analysis

Raman spectra are complex and require data analysis to illustrate detailed information of the sample that cannot be visualized by the naked eye. Therefore, collected Raman data are preprocessed to extract the characteristic band(s), followed by establishing classification or prediction models and finally applying them for the determination of analytes in real samples [13].

Data preprocessing is used to remove spectral noises, artifacts, and useless signals from raw data that are caused by test environments and flaws in imaging equipment [4]. Data processing techniques include spike correction, wavenumber calibration, intensity calibration, smoothing, background correction, normalization, and dimension reduction [14]. When selecting data processing techniques, it is important to evaluate different methods and validate the data analysis approaches; otherwise, information may be lost [4].

Multivariant classification models extract different information from Raman spectra using chemometrics to classify and distinguish samples. Based on additional prior information, these models can be divided into unsupervised and supervised pattern recognition. Unsupervised pattern recognition is used to discover hidden structures in unlabeled data. Principle component analysis (PCA), the most used unsupervised learning algorithm, reduces the dimensions of spectral data. For the classification of samples, it is widely combined with clustering algorithms. Supervised pattern recognition uses a set of samples of known categories to establish a mathematical model. This model can then classify unknown samples. Commonly used discriminant techniques are linear discriminant analysis, partial least square discrimination analysis, support vector machine (SVM), and artificial neural network. Predication models identify the relationship and influence of variables and use this information to predict a parameter of a new observation. As the selection of an appropriate mathematical model is crucial, several model result evaluation systems have been established. For example, determination coefficient (R^2^) and root mean square error (RMSE) can be applied to optimize model parameters and model performances [13].

## 3. Biological Hazards

Foods are capable of supporting the growth of microbes due to their nutrient compositions. Food products contaminated by human pathogens can lead to foodborne infections and diseases [15]. Foodborne pathogenic microbes include bacteria, viruses, fungi, and parasites. Viruses are responsible for more than 50% of foodborne illnesses, and the most common foodborne viruses are norovirus, rotavirus, astrovirus, and Hepatitis A [15]. Severe cases leading to hospitalizations and death are primarily caused by bacteria. Bacterial genera and species responsible for over 90% of foodborne illnesses are *Staphylococcus*, *Salmonella*, *Clostridium*, *Campylobacter*, *Listeria*, *Vibrio*, *Bacillus*, and Shiga-toxigenic *E. coli* [1]. Foodborne diseases are a major burden to public health worldwide. For example, the Centers for Diseases Control and Prevention (CDC) estimates that 48 million people get sick, 128,000 people are hospitalized, and 3000 people die in the United States annually due to foodborne diseases [16].

Routinely used methods for the detection of foodborne bacterial pathogens in the food chain are based on culturing bacteria in specific nutrient-rich media followed by standard biochemical identifications. These conventional culture-based methods are inexpensive, but labor-intensive, time-consuming, less sensitive, and cannot cultivate the viable but non-culturable (VBNC) state of bacteria. Furthermore, this well-established detection method shows variable specificity depending on the complexity of the food matrix and the isolation procedure [16,17]. To improve the sensitivity and specificity of culture-based methods, a variety of rapid, culture-independent molecular methods have been increasingly used as routine detection methods for foodborne pathogens. Nucleic acid-based methods identify unique, specific DNA or RNA sequences by amplification, followed by visualization on gel, or molecular typing. The most reliable and commonly used identification methods for pathogens are polymerase chain reaction (PCR) assays. One major limitation of nucleic acid-based methods is the inability to differentiate between viable and dead cells [16,17]. Other detection methods for foodborne bacterial pathogens include immunological-based methods (e.g., enzyme-linked immunosorbent assay (ELISA) and lateral flow immunoassay) and biosensors (e.g., optical, biochemical, and mass-based biosensors) [16]. Compared to bacteria, the detection of viruses in foods is more challenging because viruses cannot be enriched in media. Sensitive detection of foodborne viruses in food matrices has been achieved by molecular techniques such as PCR, microarray, biosensors, and next-generation sequencing [18].

Due to significantly increasing incidences of foodborne diseases worldwide, a rapid, specific, and sensitive detection method of foodborne microbes is needed [16]. Raman spectroscopic methods have the potential to be applied in the agri-food industry, and efforts have been made towards improving current and developing new Raman-based detection methods.

### 3.1. Determination of Foodborne Bacteria

Several studies have used micro-Raman spectroscopy to differentiate between foodborne bacteria and confidently identify pathogens to at least species level. One study identified 16 staphylococcal species directly from bacterial colonies grown on an agar plate with an almost 100% success rate by using a model comprising characteristic Raman spectra of 277 staphylococcal strains [19]. Successful differentiation of *Arcobacter* from the closely related bacterial genera *Campylobacter* and *Helicobacter* was achieved using confocal micro-Raman spectroscopy and PCA. Furthermore, 82 *Arcobacter* strains representing 18 *Arcobacter* species were discriminated to the species level with an accuracy of 97.2% using a convolutional neural network. In the same study, *Arcobacter* species ratios in a bacterial mixture ranging from 5% to 100% were predicted with a high linear correlation between the actual and the predicted ratio (regression coefficient >0.99) by a Raman spectroscopy-based fully connected artificial neural network [20]. Jaafreh and co-authors achieved the discrimination and classification of eight important bacterial strains (e.g., strains of *Bacillus*, *Pseudomonas,* and *E. coli*) commonly found in poultry meat with an accuracy up to 100% by using two dispersive Raman spectrometers (Microscope and Portable Fiber-Optic systems). Discrimination and classification models were based on PCA and multi-class SVM [21]. Another study also showed the differentiation of meat spoilage microorganisms to genera and strain level by micro-Raman spectroscopy combined with PCA with an error rate of 3.5%. The direct and fast spectral collection was performed by rapid surface blots that require no pretreatments such as purification or singulation steps [22]. Another study collected 15,890 single-cell Raman spectra of 23 common strains from seven genera (i.e., *Escherichia*, *Listeria*, *Staphylococcus*, *Cronobacter*, *Vibrio*, *Shigella,* and *Salmonella*) with a confocal Raman microscopic system. The data were processed by Kernel PCA combined with decision tree (KPCA-DT). KPCA extracted the nonlinear features of raw data, while the DT algorithm discriminated the bacterial cells by hierarchical classification models. The identification of strains at the serotype level was achieved with accuracies in the range of 87.1–95.8% [23]. Successful differentiation of *E. coli*, *B. cereus*, *S. aureus,* and *S. typhimurium* on an aluminum slide was demonstrated by Raman scattering with near-IR wavelength excitation coupled with multivariate analysis. The advantage of using an aluminum slide compared to a gold/silver slide or SERS-substrate is that it reduces the detection cost [24].

**Figure 4:**
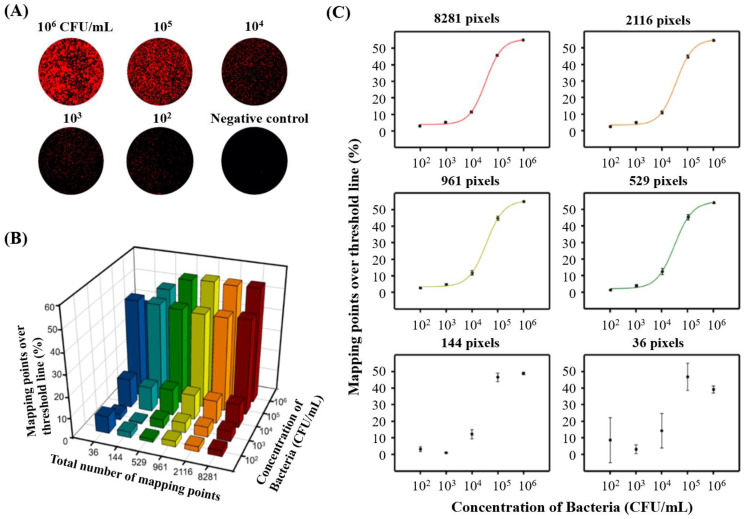
Quantitative evaluation of *Salmonella enterica* serovar *S. typhimurium* by SERS imaging. (**A**) SERS mapping images measured with a peak intensity at 1615 cm^−1^ for different concentrations of *S. typhimurium*. (**B**) Mapping points over the threshold line (%) in the 0 to 10^6^ CFU/mL range. (**C**) Corresponding standard calibration curves as a function of bacteria concentration for different numbers of total mapping points [28].

Besides Raman spectroscopy, several studies applied SERS for the discrimination of bacteria. For example, a study detected and discriminated *E. coli* O157:H7, *S. aureus,* and *Salmonella* characteristic peaks in SERS spectra with PCA and hierarchical cluster analysis. Their assay used silver colloidal nanoparticles as highly sensitive SERS-active substrates and had a detection time of a few minutes [25]. Another study differentiated seven important meat-associated microorganisms (e.g., strains of *Salmonella*, *Listeria,* and *E. coli*) at genus level by using commercial paper-based SERS substrates covered with gold nanoparticles with an independent test error of 2.5%. SERS spectra were pre-processed with spike correction and sum normalization followed by classification with subsequent chemometric evaluation (i.e., PCA and canonical discriminant analysis) [26]. Furthermore, the differentiation and quantification of pathogens were achieved by SERS mapping. For instance, *S. enterica* and *E. coli* were detected and identified from a mixture sample simultaneously using SERS mapping and PCA. This developed label-free SERS mapping method used silver dendrites to collect spectra. This method revealed a LOD as low as 10^4^ CFU/mL, and the mapping time for a 225 points map was approximately 24 min [27]. Quantification of *S. typhimurium* was achieved with SERS mapping by Ko and co-authors (Figure 4). This SERS imaging method was performed by a three-dimensional (3D) silver/gold core-shell nanopillar substrate and works as follows. After bacteria were immobilized on the positively charged poly(L-lysine)-coated 3D plasmonic substrate through electrostatic interactions, the bacterial surfaces were selectively labeled with antibody-conjugated SERS nanotags. SERS nanotags appeared as red dots, while other areas appeared dark on Raman mapping images. These Raman images revealed that an increase in the bacterial concentration led to an increase in the number of red dots (Figure 4A). A statistically reliable standard calibration curve relating bacterial concentration and different numbers of total mapping points was retrieved at 529 pixels (Figure 4B,C), which required a total mapping time of 45 min. This SERS mapping method did not require any pre-enrichment and detected concentrations of *S. typhimurium* as low as 10^2^ CFU/mL (Figure 4B) [28].

### 3.2. Detection of Bacteria in Different Survival Stages

Most microorganisms develop a form of survival mechanism to cope with stress conditions. Biofilms have been associated with many foodborne outbreaks and are estimated to cause 80% of persistent bacterial infections in the United States [29]. Biofilms are an aggregation of microbial cells surrounded by a matrix of extracellular polymeric substances and adherent to inert or living surfaces. Unlike planktonic single cells, biofilms provide the encased cells a protective barrier, and therefore, it is regarded as a virulence factor. Biofilm cells have shown enhanced resistance to antibiotics, disinfectants, adverse environmental conditions such as extreme temperatures and pH, and host defenses [29,30]. Identification and detection of biofilms of foodborne pathogens have been demonstrated using Raman spectroscopy and SERS. Determination among nine bacteria (e.g., species of the *Legionella* genus, *Pseudomonas aeruginosa,* and *E. coli*) grown as both planktonic cells and biofilms in tap water was achieved by micro-Raman spectroscopy combined with SVM [31]. Liu and co-authors reported a rapid and novel SERS detection method to study *Staphylococcus aureus* biofilm formation. Raman spectra of crystal violet-stained biofilms were collected using a portable Raman spectrometer with gold nanoparticles. Measured Raman peak intensities exhibited a good linear relationship with the amount of biofilm [32]. Another study used Fourier transform infrared (FTIR) spectroscopy and Raman spectroscopy to distinguish between planktonic and biofilm cells of two isolates of *Cronobacter sakazakii*. Raman spectra revealed that biofilms had higher intensity in the bands assigned to tyrosine, amide III, carbohydrates, carotenoids, DNA, and lipids, while FTIR spectra of biofilms exhibited a higher intensity in the bands assigned to polysaccharides, amide I and amide II. These spectral features of both spectroscopic methods aided in identifying differences in the cellular composition of planktonic and biofilm cells [33]. Characterization of composition and structure of biofilms by Raman spectroscopy and SERS is summarized in another review paper [34].

The viable but non-culturable (VBNC) state is another survival strategy of microbes against harsh environmental stresses, such as nutrient starvation, extreme temperatures, osmotic stress, oxygen availability, chemicals (e.g., chlorine and ethanol), and exposure to white light. The VBNC state is defined as a state of dormancy in which bacterial cells still exhibit active metabolism but cannot be cultured on the routine bacteriological media and thus evade routinely used culture-based methods. Bacteria in the VBNC state can remain dormant for several years but are believed to not generate any infections and/or diseases. However, bacteria in the VBNC state can resuscitate under favorable conditions, regain virulence, and subsequently cause infections [35]. Bacteria in the VBNC state have been identified based on their reduced metabolic activity by Raman spectroscopy. Only a few limited studies of identifying VBNC foodborne bacteria by Raman spectroscopy and SERS have been reported to date. One of them detected UV-induced VBNC bacteria (i.e., one strain of *Aeromonas* spp., *Pseudomonas* spp., *E. coli,* and *S. aureus*) at both population and single-cell levels by heavy water (D_2_O)-labeled confocal micro-Raman spectroscopy. As the deuterium isotope of hydrogen can substitute hydrogen in water and form C-D bonds during bacterial synthesis of fatty acids and proteins, reduction of bacterial metabolic activity was determined according to the decrease in Raman intensity of C-D bands in the region of 2040–2300 cm^−1^. With an increase of UV dosage from 10 to 200 mJ/cm^2^, the C-D bands were reduced by 95.7% and 47.9%, respectively, compared to the unirradiated controls, depending on the strain and UV dose. The single cellular Raman spectrum detected VBNC bacteria by their metabolic activity reliably and showed a heterogenic distribution of metabolic activity in VBNC bacteria [36]. Another study used the same approach for detecting anaerobic stress-induced VBNC *Rhodococcus biphenylivorans* by single-cell confocal micro-Raman spectroscopy combined with D_2_O. They also observed a significant decrease in metabolic activity once bacterial cells entered the VBNC state [37]. Fu and others also reported the differentiation of culturable, VBNC, and dead cells of *E. coli* O157:H7 by D_2_O-labeled confocal micro-Raman spectroscopy [38]. It is promising that Raman spectroscopic methods can detect bacteria in various survival stages (e.g., VBNC state and biofilms) as those are a severe concern to food safety and public health [29,35]. However, generic detection of pathogens in multi-biofilms and mixtures of dead, culturable, and VBNC bacteria has not yet been shown by Raman spectroscopic methods.

### 3.3. Detection of Bacteria in Drinking Water

**Figure 5:**
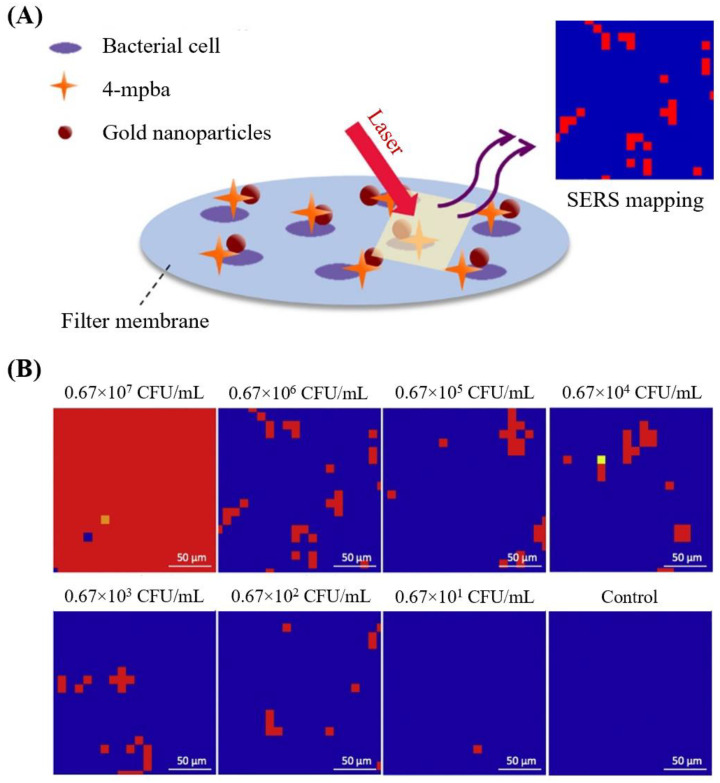
Quantitative SERS detection of pathogens using 4-mpba as capture and indicator combined with filtration. (**A**) Schematic illustration of the bacterial detection by SERS mapping; (**B**) mapping images of *E. coli* cells at different concentrations [45].

Clean and safe drinking water is essential to life, but many people still have limited access to a safe water supply in developing countries. Developed countries are also affected by waterborne diseases. It is estimated that waterborne infections cause 560,000 severe diseases and 12,000 deaths in the United States annually [39]. Determination of waterborne pathogens at low concentrations was achieved by Raman spectroscopy and SERS. For example, one study detected *E. coli* O157:H7 in water in the presence of non-target interference with an accuracy above 95% using PCA and SVM. Their developed biosensor combined a nano-dielectrophoretic microfluidic device and multiplexing dual recognition SERS based on two noble metal nanoparticles. The limit of detection was determined to be 1 CFU/mL (single-cell level) [40]. Another study discriminated three strains of *E. coli* and one strain of *Staphylococcus epidermidis* in drinking water by label-free SERS mapping of bacteria with silver nanoparticles synthesized on the cell wall. The sensitivity of this assay was determined to be 2.5 × 10^2^ cells/mL [41]. Silge and co-authors identified strains of *P. aeruginosa* from non-*P. aeruginosa* strains in different mineral waters using micro-Raman spectroscopy combined with filtration and fluorescence staining of bacterial cells. Their reference dataset of single-cell Raman spectra accounted for various parameters, such as growth stages of strains and four types of water with different mineral contents and pH, improving the identification performance of the model by PCA and SVM. The model predicted *P. aeruginosa* and non-*P. aeruginosa* correctly even when the growth conditions were not the same as those in the reference database. Testing the detection of *P. aeruginosa* and non-*P. aeruginosa* in four water types revealed that the model can handle new spectral characteristics within the range of the spectral properties of the model but not in waters with more extreme mineral and pH content than the reference samples. The total detection time of the assay was 2–3 h for 50–100 representative single cells per sample, and tested samples contained approximately 10 CFU/mL [42]. Another study used silver-coated magnetic nanoparticles to capture *Acintobacter baylyi* and *E. coli* in drinking water for the detection by SERS. This detection method showed a sensitivity of 10^5^ CFU/mL and achieved rapid screening of bacteria in water in less than 15 min [43]. Krafft and co-authors developed a microfluidic device that trapped and concentrated bacterial cells of *E. coli* and *Pseudomonas* as well as aggregated silver nanoparticles from tap water by the electrokinetic flow of the sample across a porous membrane. Then, SERS spectra of pathogens captured on polycarbonate membrane were collected. This developed lab-on-chip device detected pathogens in tap water rapidly and cheaply [44]. Detection of *E. coli*, *S. enterica*, and *L. monocytogenes* was achieved by a combination of filtration, 4-mercaptophenylboronic acid (4-mpba), and SERS mapping (Figure 5A). For this assay, bacterial cells were captured on a filter membrane, and then a 4-mpba solution was added to specifically bind 4-mpba to the surface of bacteria. After non-bond 4-mpba were washed out, gold nanoparticles were filtered onto the membrane and SERS images were scanned directly from the membrane. SERS mapping at different bacterial concentrations revealed that the percentage of positive points (red in Figure 5B) can estimate the number of total bacteria cells present in the sample. This assay took approximately 1.5 h and was applied for pond water analysis [45]. However, it also has the potential to be used for drinking water or even food samples.

### 3.4. Detection of Bacteria in Foods

In most food samples, the target pathogen exists along with a competing microflora in a complex background matrix. Ideally, a detection method suitable for the food industry detects viable cells of the target microbe, specifically in a rapid, reproducible, cheap, easy-to-use, and sensitive manner [46]. The specific, rapid, and sensitive detection of pathogens in complex and heterogeneous food matrices has not been very successful by Raman spectroscopic methods as the separation between target bacteria and background is either challenging or time-consuming [47].

Here, we will introduce recent approaches that detect pathogens in both liquid and solid foods using Raman spectroscopy or SERS. One study distinguished six *Listeria* species in milk by confocal micro-Raman spectroscopy coupled with chemometric models, including PCA and hierarchical cluster analysis with a classification accuracy of over 93%. Although this detection method can be finished in a few hours, it requires a time-consuming enrichment step before sample preparation unless the initial concentration is very high (~10^8^ CFU/mL) [48]. A faster approach to detect pathogens in milk is to integrate a 4-mpba functionalized silver dendritic substrate with SERS mapping as it concentrates, detects, and differentiates bacteria in one step. This method is similar to the assay above that detects *E. coli*, *S. enterica,* and *L. monocytogenes* using 4-mpba and gold nanoparticles (Figure 5), except that the capturing of bacteria by 4-mpba is done in solution and not on a filtration membrane. SERS mapping with 4-mpba functionalized silver dendrites detected *S. enterica* in skimmed milk with a limit of detection of 10^2^ CFU/mL. The time to collect a mapping image containing as many as 400 individual spectra took 28 min and another 30 min was needed for sample preparation beforehand [49]. SERS was also used to detect pathogens in fruit juices. In one study, *E. coli* O157:H7 was separated and concentrated by capture antibodies (cAbs) that were immobilized on magnetite–gold magnetic nanoparticles (MNPs). Capture efficiency for *E. coli* O157:H7 by MNPs was determined to be approximately 84–94%. For the SERS detection, gold Raman labeled detector antibodies (dAb) interacted with gold-coated MNP-cAb-*E. coli* O157:H7 complex. This assay detected bacterial cell concentrations as low as 10^2^ CFU/mL in apple juice in less than 1 h. Additionally, they observed no cross-reactivity with non-target organisms when present in background, and a linear relationship between Raman intensity and bacterial concentration was observed, indicating quantitative capacities [50].

Bacterial pathogens in solid food products can be determined by isolation and enrichment of bacteria in nutrient media followed by the detection using Raman spectroscopy or SERS. Some studies have focused on the development of standardized procedures. One study developed a method to detect *S. enterica* in rice, oat, wheat, maize, chicken, and pork by micro-Raman spectroscopy and chemometrics with the purpose to establish it as an ISO method. As they proposed an isolation and enrichment procedure, they accounted for the physiological state and the growth phase of microbial species when Raman spectra were collected, making it a more reliable and reproducible method [51]. Another study had a similar goal to introduce the SERS technique into IOS standard procedures. Therefore, they demonstrated the detection of *L. monocytogenes* and *Salmonella* spp. in smoked salmon, *L. monocytogenes* in ham, *Salmonella* spp. in eggs, and *Cronobacter* spp. in powdered infant formula and mixed herbs by SERS microscopy based on silver nanoparticles combined with PCA. They also introduced isolation and enrichment steps before SERS detection [52]. Although these methods were able to reduce the detection time significantly from approximately 6 to 2 days [51,52], it is still a time-consuming and labor-intensive detection approach.

Faster detection of pathogens in food products can be achieved by direct detection on the food surface. For example, one study monitored microbial counts of beef steaks stored under two different packaging methods (e.g., vacuum and modified atmosphere packaging) at 4 °C for up to 21 days by comparing Raman spectroscopy combined with partial least square regression to the plating assay. Raman spectra collected directly from the steak surface demonstrated the ability to predict total viable counts and lactic acid bacteria in beef for both packaging methods at days 0 and 21. This study showed the potential to rapidly determine meat spoilage without having to remove the meat from the packaging by Raman spectroscopy [53]. Another study investigated the time-dependent changes of chicken meat to detect spoilage by combining FTIR and Raman spectroscopies, similarly to the study above that differentiated planktonic from biofilm cells. They successfully demonstrated that chicken meat spoilage can be detected by both its metabolic change and effects of the microbial loads in the food samples using Raman and FTIR spectra combined with deconvolution of the experimental bands into Lorentz components. Raman spectra revealed the decrease in protein content (i.e., bands at 1655 and 1320 cm^−1^) and the increase in amino acids (i.e., band at 1675 cm^−1^) during spoilage within 10 days [54].

### 3.5. Detection of Non-Bacterial Pathogens

Besides the detection of bacteria, Raman spectroscopic methods have also been developed for the detection of viruses and fungi to improve food safety. Detection of norovirus was achieved by a SERS immunoassay. Norovirus was captured and separated by antibody-mag-MoO_3_ nanocubes, followed by attachment of this complex to SLGO-4MBA-antibody capture substrate for SERS detection. The limit of detection of this method was determined to be ∼60 RNA copies/mL in fecal samples, which is ∼10^3^-fold more sensitive than routinely used commercial ELISA kit for norovirus [55]. Applications of this method in food products may also yield promising results. Other studies showed the detection of fungi by SERS as fungi cause spoilage and thus are a potential threat to the public health and economy [56,57]. A total of five fungi associated with spoilage in apples were distinguished by SERS based on gold nanorod substrate combined with PCA and linear discriminant analysis with a discrimination accuracy of 98.31% [57]. In another study, *Alternaria alternata* was detected in pear juice by SERS-based silver nanodots array. Different concentrations of spiked pear juice were directly added onto the SERS substrate and scanned by Raman mapping. The lowest detectable *A. alternata* concentration in pear juice was less than 10^4^ CFU/mL, and a correlation between the Raman intensity and fungi concentration was determined [56].

## 4. Chemical Hazards

While biological food safety hazards are often the focus of food companies and regulators, more attention has been paid to chemical hazards in the past decades [58]. Chemical hazards are substances that are present in foods at levels that can be harmful to humans. These compounds can either occur naturally (e.g., toxins, heavy metals, allergens) or be intentionally added to food products (e.g., pesticides, additives) [59]. A representative example of the importance of controlling chemical hazards is the deliberate contamination of infant formula with melamine that occurred in China in 2008 and affected 300,000 infants and young children, of whom 51,900 were hospitalized and six died [1].

The most routinely used detection method for chemical compounds is high-performance liquid chromatography (HPLC) or gas chromatography for separation combined with ultraviolet or mass spectrometry (MS) for detection. Although these methods are robust and reliable, they are time-consuming and expensive, require highly trained personnel, consume large amounts of chemical reagents, and fail to realize a high-throughput screening [59,60]. ELISA is the most employed detection method of allergens by the food industry and official food control agencies as it is fast, reliable, and user-friendly and allows easy and simultaneous analysis of several samples. However, the sensitivity highly depends on the extractability of allergens from the food matrix [61].

Raman spectroscopy is a promising and emerging method to detect chemicals in food as it is a non-destructive, rapid, specific, ultrasensitive, and high-throughput screening method. Additionally, portable Raman detection systems are available and make on-site detection possible compared to HPLC–MS [59,60].

### 4.1. Detection of Mycotoxins

Mycotoxins are naturally occurring toxic compounds produced by certain fungi. These fungi can grow on foods (e.g., cereal, spices, nuts, dried, and fresh fruits) before or after harvest, during storage, and on/in the food itself. Foodborne mycotoxins cause a variety of severe illnesses including acute poisoning and long-term effects such as immune deficiency and cancer. Although hundreds of mycotoxins have been identified, only a few dozen (e.g., aflatoxins, zearalenone, and deoxynivalenol) cause severe effects on human health. International standards and codes of practice have been established to limit the presence of mycotoxins in certain foods. For example, 0.5‒15 µg/kg of aflatoxins has been determined to be the maximum level in various nuts, grains, dried figs, and milk by Codex Alimentarius Commission [62].

Raman spectroscopy combined with multivariate algorithms (i.e., synergy interval partial least squares and ant colony optimization) successfully quantified zearalenone contents in ground maize without sample-extraction steps. In this study, 85 maize samples naturally contaminated with zearalenone concentrations ranging between 6.9–800.2 μg/kg were detected by Raman spectroscopy, and results were validated by HPLC [63]. Another study quantitatively detected deoxynivalenol spiked in pig feed by using SERS based on core-shell silver nanocubes coated with polydopamine. The limit of detection of this developed assay was determined to be 0.82 × 10^−15^ M, and a linear relationship between SERS intensity and the logarithm of deoxynivalenol concentrations was seen with an R^2^ of 0.9958 [64].

### 4.2. Detection of Environmental Contaminants

Heavy metals are naturally occurring elements in the soil, water, and atmosphere. Metals can enter the food supply chain directly through the environment, by human activities (e.g., farming, industry, or car exhausts), or from contamination during food processing and storage. Some heavy metals can be beneficial to health and are intentionally added to certain foods (e.g., iron), while other heavy metals have adverse health effects (e.g., arsenic, cadmium, lead, and mercury) [65,66]. For example, mercury is a severe threat to the safety of organisms as it has high toxicity and can cause paralysis and deformity. Bao and co-authors detected mercury ions in tap water using SERS and gold-film-supported organometallic nanobelts. This assay was based on the formation of ultrafine HgS nanoparticles upon the reaction of mercury ions and nanobelts. As the nanobelts are coated on a SERS active gold nanoparticle film, HgS can be detected by SERS. The detection limit was determined to be at ppt level, which is two orders of magnitude lower than the mercury toxicity level defined by the US Environmental Protection Agency [67]. Another study detected cadmium ion in three types of rice using SERS based on gold nanoparticles modified with trimercaptotriazine. Cadmium ion is one of the most toxic heavy metal ions as it causes serious damage to organs such as kidneys, liver, and lungs. In this method, trimercaptotriazine chelates with cadmium ions and simultaneously binds to gold nanoparticles. The changes induced by the chelation of trimercaptotriazine with cadmium ions can be detected by SERS. This developed method showed a limit of detection of 8 μg/kg and a limit of quantification of 24 μg/kg [68].

Bisphenol A (BPA) is a compound used to produce polycarbonate plastics, epoxy resins, coatings of food or drink packages, and other packing materials. Although it is ubiquitous in our daily lives, it is harmful to human health once released from packaging materials. It has been shown that BPA can interfere with the endocrine system and potentially lead to increased cell proliferation of the male and female sexual organs. BPA was detected in bottled water using a SERS-based competitive immunoassay. In this assay, SERS-labeled antibodies were able to bind to either BPA antigens or coated antigens immobilized on substrate. If no BPA were present, the SERS-labeled antibodies would be captured by coated antigens, giving off a strong SERS intensity. In contrast, if high amounts of BPA were present, the SERS-labeled antibodies would be captured by BPA antigens and not attach to the substrate. Consequently, no or a weak SERS signal would be detected after washing. This assay quantitatively detected BPA by relating the intensity of the SERS signal and the concentration of BPA. The detection limit of BPA was determined to be 1 ng/mL in water [69]. More studies showed the detection of other environmental pollutants from food contact materials by micro-Raman spectroscopy and a method that combined thin-layer chromatography and SERS [70,71].

### 4.3. Detection of Additives and Adulteration

**Figure 6:**
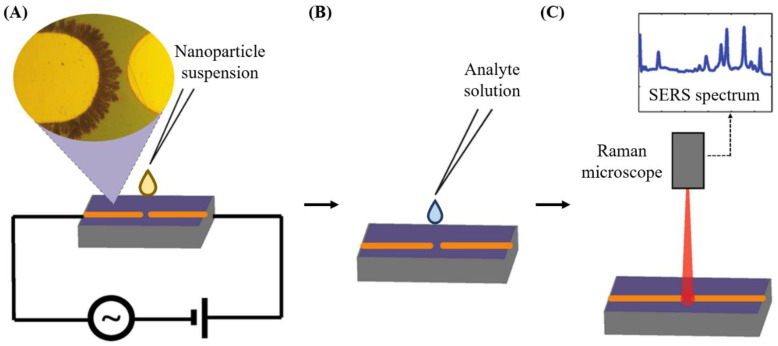
Preparation of SERS substrate used for the detection of food contaminants in liquid food. (**A**) Formation of SERS-active nanostructures. (**B**) Addition of analyte solution to the surface of the microelectrodes. (**C**) SERS detection of the analyte [60].

Food additives are substances derived from plants, animals, or minerals, or are produced synthetically and are added intentionally to the food to maintain and/or improve its safety, freshness, taste, texture, or appearance. Before additives are approved for use in foods, they are assessed for their potentially harmful effects on human health. Most food additives have maximum use levels and must be indicated on the label in most countries [72]. Raman spectroscopic methods can be applied for the detection of food additives. For example, SERS based on a flower-like silver-based substrate coupled with intelligent multivariate analysis quantitatively detected coumarin with a detection limit of 1.46 μg/kg. Coumarin is a natural ingredient that smells like fresh hay and vanilla and thus was originally used as a flavoring agent in food and tobacco. Today, coumarin is banned as a food additive in most countries because it was found to have hepatotoxic effects on rats and might be linked to cancer [73]. Another study detected the oxidizing agent potassium bromate in unbleached all-purpose wheat flour using a line-scan hyperspectral Raman imaging system. They demonstrated non-targeted detection of potassium bromate in a background of unknown contaminants (i.e., azodicarbonamide, benzoyl peroxide, and L-ascorbic acid) using mixture analysis algorithms followed by a spectral matching method [74]. The detection of melamine (false increase in protein concentration), sodium thiocyanate (preservative), and lincomycin hydrochloride (antibiotic) in milk were achieved by single-drop Raman imaging that incorporates the coffee-ring effect for sample pretreatment and discrete wavelet transform for spectra processing. The sensitivities of this assay for sensing melamine, sodium thiocyanate, and lincomycin hydrochloride were determined to be 0.1 mg/kg, 1 mg/kg, and 0.1 mg/kg, respectively [75]. Detection of melamine by a Raman spectroscopic method was also demonstrated by another study. They used SERS based on a dendritic silver nanostructure to detect melamine in milk and infant formula. The SERS substrate was produced on the surface of a microelectrode chip (Figure 6). SERS-active nanostructures were formed when gold microelectrodes were connected to the mixed AC/DC voltage supply and the nanoparticle solution was sitting at the tips of the microelectrode (Figure 6A). Then, analyte solution was placed upon the sensing surface (Figure 6B), and finally, a SERS spectrum was obtained by analyzing the gap region between two microelectrodes (location of both the SERS active structures and the analyte) with a Raman microscope (Figure 6C). The detection limits of this assay were 1.5 ppm in milk and 105 ppb in infant formula. These levels were below limits recommended by the US-EPA for milk and WHO for infant formula [60].

Adulteration is the act of intentionally lowering the quality of food by either adding or substituting inferior substances or by the removal of some valuable ingredient. Adulterants are commonly applied to improve the appearance of food or increase economic gain [76]. Besides adulteration causing image loss of food products due to dishonesty and depriving consumers of vital nutrients, adulterants can be hazardous to human health as they may be toxic or induce allergies [77]. Examples of adulterations that can be detected by Raman spectroscopic methods include olive oil adulterated with rapeseed and corn oil [78], honey adulterated with fructose, glucose, inverted sugar, hydrolyzed inulin syrup, and malt must [79], pistachio nuts adulterated with green peas [80], cassava starch adulterated with wheat flour or sodium bicarbonate [77], and butter adulterated with margarine [81].

### 4.4. Detection of Pesticides

Pesticides are used in agriculture worldwide to control pests and improve the growth of plants [82]. Besides facilitating the increase in the yield, pesticides are hazardous chemicals. They can have an adverse impact on human health and wildlife and have been shown to be linked to diseases such as cancer, hormone disruption, asthma, and allergies [83]. Several studies have demonstrated the ability of Raman spectroscopic techniques to detect pesticides in food. For example, thiram, a fungicide for fruit, was detected in apple juice by the same aforementioned SERS-based method that detected melamine in milk and infant formula (Figure 6). The limit of detection was determined to be 115 ppb with no sample preparation [60]. In situ detection of thiabendazole, another fungicide for fruit preservation, was achieved by SERS and a gold nanoisland tattoo. Therefore, a tattoo paper with a water-soluble release layer was coated with a gold island film and transferred onto the surface of oranges spiked with different concentrations of thiabendazole. Obtained SERS spectra revealed a detection limit of 0.2 ppm, which is below the EU-specified maximum residue levels. Furthermore, this assay distinguished thiabendazole from soybean oil in a mixed sample, showing the capability of multiplex detection [84]. Another study also detected thiabendazole in food by using SERS based on a silver nano-substrate combined with chemometric methods (e.g., partial least squares). This assay reliably detected five rape samples with unknown thiabendazole concentration, as results were comparable to UPLC. The limit of detection was determined to be 0.1 mg/L, but pretreatment of samples was required [85]. Simultaneous detection of thiram and thiabendazole on the surfaces of apple, tomato, and pear was achieved by SERS coupled with interfacial self-assembly gold nanorods array substrates. Spiked pesticides on fruit were extracted with a modified surface swab method followed by the collection of SERS spectra and their analysis using self-modeling mixture analysis. The detection limits of the pesticides on the surface of apple, tomato, and pear were 0.041, 0.029, and 0.047 ng/cm^2^ for thiram, and 0.79, 0.76, and 0.80 ng/cm^2^ for thiabendazole, respectively [86]. Another study detected carbendazim, a fungicide, in Oolong tea using SERS based on spherical and monodispersed gold nanoparticles. Spectral data analysis was performed by partial least squares analysis and leave-one-out cross-validation. Sensitivity was determined to be 0.1 ppm, but pretreatment of samples was required [87]. Other studies focused on the detection of organophosphate insecticides. For instance, chlorpyrifos was determined on tomato surface and in tomato extract by SERS based on silver colloid substrates. The detection limit of this assay was 10^−9^ mol/L, which is below the standards of chlorpyrifos in China, Japan, and the EU. Linear correlation between chlorpyrifos concentrations and SERS intensities at characteristic peaks were observed over the range from 10^−3^ to 10^−9^ mol/L. Although no pretreatment was required for the detection of chlorpyrifos on the tomato surface, sample preparation of tomato extract was needed [88]. Yaseen and co-authors used a similar method (i.e., SERS based on silver colloid substrate) to detect another organophosphate insecticide (i.e., omethoate) in peach extract. Their limit of detection was determined to be 0.01 mg/kg [89]. Detection of two insecticides (i.e., pymetrozine and thiamethoxam) and one herbicide (i.e., 2,4-dichlorophenoxyacetic acid) was demonstrated by SERS based on compactly arranged gold nanoparticles templated from mesoporous silica film. A linear relationship was observed between the concentration of pesticides and SERS intensity. Detection limits for 2,4-dichlorophenoxyacetic acid of 0.79 pg/mL, pymetrozine of 1.04 pg/mL, and thiamethoxam of 1.21 pg/mL were achieved within the linear ranges of 0.1–1000 ng/mL. Determination of 2,4-dichlorophenoxyacetic acid was also successfully shown in tap water, apples, and milk using sample preparation methods and the developed SERS assay [82]. A total of 21 pesticides, including fungicides and insecticides, were detected using SERS based on colloidal gold nanoparticles and PCA. The 21 pesticides showed limits of detection ranging from 0.001 to 10 ppm. Furthermore, they demonstrated the simultaneous detection of phosmet and thiram at different concentrations (i.e., ratios of 1:3, 1:1 and 3:1) in both a mixture solution and on apple skin [83].

### 4.5. Detection of Allergens

A food allergy is defined as an adverse reaction to food involving an immunological mechanism against a certain allergen. Individuals with food allergies usually experience mild to acute symptoms affecting the skin, gastrointestinal tract, respiratory tract, eyes, and/or central nervous system but rarely die. Common foods that trigger severe allergies include cereals containing gluten, crustaceans, eggs, fish, peanuts, soybeans, milk, and tree nuts [90]. A few studies have been conducted on the detection of allergens by Raman spectroscopic methods. One study showed that Raman spectroscopy combined with PCA can distinguish lactose-free and regular milk samples based on the band at 355 cm^−1^. However, this assay did not investigate the detection of specific milk allergens [91]. For the detection of allergens, methods combining an immunoassay and Raman spectroscopy were developed. For example, a peanut allergen protein (i.e., Ara h1) was detected by a biodegradable gold/zein film SERS platform and PCA. To improve the specificity, Ara h1 was captured with monoclonal antibodies. The detection limit of this assay was determined to be 0.14 mg/mL [92]. Another study developed a SERS-based lateral flow immunoassay strip to detect the soybean allergen *β*-conglycinin. The limit of detection of these double antibody sandwich strips was 1 μg/mL and visible with the naked eye upon coloration. For quantification, the Raman intensity of *p*-aminothiophenol with colloidal gold was measured. The quantitative range of this assay was between 100‒160 ng/mL of *β*-conglycinin [93].

## 5. Physical Hazards

Physical hazards are extraneous materials that are not commonly found in foods, such as metal, glass, plastic, wood, rocks, and insects. They are considered hazardous because their hardness, sharpness, size, or shape may cause lacerations, perforations, wounds, and choking [94]. Detection methods employed to control physical hazards are diverse and depend on the material. Strategies include visual inspection, metal detection, magnets, optical and laser sorters, X-ray technology, screens, filters, and sieves. Companies also establish a hazard analysis critical control point (HACCP) plan and good manufacturing practices (GMP) to control physical hazards [95].

Plastic particles smaller than 5 mm but larger than 0.1 µm have been defined as microplastics [96]. Microplastic is ubiquitous in the environment including foods and beverages and can be a threat to the environment and human health [97,98]. Plastic and large microplastics are often determined by visual sorting, filters, and sieves. Identification of microplastic particles from complex matrices can be challenging due to their various sizes, shapes, and polymer types. Commonly, microplastic analysis involves physical identification of plastics (e.g., microscopy) and chemical characterization (e.g., spectroscopy, thermal analysis) for confirmation of plastics [98]. Here, we will introduce some recent studies that used Raman spectroscopic methods to detect nano- and microplastics. Detection of polystyrene and polymethyl methacrylate microplastics with sizes down to 360 nm was achieved by SERS combined with Klarite substrates. Klarite substrates are a dense grid of inverted pyramidal cavities made of gold that create intense hotspots. Furthermore, Raman mapping combined with this SERS approach can be a fast and consolidated detection method of microplastics extracted from the atmosphere [97]. Another study identified five different high-production-volume polymers (i.e., nylon, polyethylene, polypropylene, polystyrene, polyethylene terephthalate) in microplastic by using stimulated Raman scattering (SRS) microscopy. SRS is based on the interaction between the photon energy difference of two laser beams and vibrational levels in the molecules of the sample. With this approach, the authors demonstrated the detection and identification of 88 microplastics corresponding to 12,000 particles/kg dry weight in an environmental sample [99]. Microplastic contamination of water has attracted great attention, and thus, detection methods to monitor microplastic particles in tap and environmental waters have been developed. For example, microbeads or plastic fragments consisting of five different polymers (i.e., polyamide, polyethylene, polymethyl-methacrylate, polystyrene, and polypropylene) were detected in tap water with a flow rate of 1 L/h in a background of fluorescence and other small particles by Raman spectroscopy. This method demonstrated the capability of monitoring microplastics smaller than 0.1 mm in streaming tap water and clear surface waters [96]. Identification of microplastics in water was also achieved by Lv and co-authors. They determined nano- and micro-plastics consisting of polystyrene, polyethylene, and polypropylene with particle sizes ranging between 100 nm and 10 μm in pure water and seawater using SERS based on silver colloid substrates. This developed method detected 100 nm plastic particles and showed a detection limit of 40 μg/mL [100]. Detection of microplastics in liquid foods was investigated by another study. This study determined microplastics in white wines capped with synthetic stoppers by micro-Raman spectroscopy. They identified at least one microplastic in 24 out of 26 wine bottles [101]. To date, limited studies have been conducted on Raman-based detection of nanoparticles consisting of plastic or other materials.

## 6. Outlook

Raman spectroscopic methods are an accepted analytical tool to evaluate food safety and have been applied for the detection of chemicals and microbes in a vast range of foods. Nevertheless, Raman spectrometers are rarely used as a high-throughput identification instrument in laboratories due to several challenges. One major drawback is the dependency on reference analytics. Most reference databases are specific to the analyte and work group. Thus, Raman spectra are collected under different conditions, such as detection method (e.g., Raman spectroscopy, SRS microscopy, and SERS), technical parameters (e.g., laser wavelength and power, integration times and spectrometer parameters), state of analyte (e.g., cultivation age of bacteria, chemical composition, and background matrix), and sample preparation (e.g., isolation procedures, analyte concentration, and different SERS substrates) [6]. To achieve fast and reliable detection, a Raman fingerprinting database that collects standardized Raman spectroscopic data from research institutions worldwide is required. Such a database would enable the direct detection of unknown analytes by matching their spectra with reference spectra in the database. Furthermore, data analysis of Raman spectra also needs to be standardized to ensure comparability of results and obtain a validated method for industrial applications as a huge variety of algorithms and models is used today [102]. More emphasis should also be put on testing developed methods on real-world food samples to evaluate their true performance, reproducibility, and robustness. Ideally, a method should detect an analyte in a variety of liquid, solid, and heterogenous foods to make it more applicable to the food industry. As Raman spectroscopy is not a separation technique, it may be necessary to develop a standard method that combines a Raman spectroscopic method with a separation technique [59]. Such an example is to integrate molecularly imprinted polymers [103] with SERS to develop an integrated sensor for the detection of various hazards in agri-food products, such as atrazine in apple juice [104]. An automated system of a standardized method that detects various analytes in food will be needed to make Raman spectroscopy a widely applied analytical tool in the food supply chain.

A major challenge of SERS measurements is caused by the inhomogeneous distribution of hot spots. A hot spot is a spatially confined region (e.g., gaps between two nanoparticles in close proximity) that exhibits an extremely high electric field enhancement and thus produces a strong SERS signal [105,106]. If hot spots are not uniformly created on a SERS substrate, it will lead to significant variations in SERS signals upon measurement. To overcome this issue, a SERS substrate that produces a “hot surface” rather than a “hot spot” is required [106].

For future studies, simultaneous detection of microbes and chemicals should be explored, as one of the major advantages of Raman spectroscopy is the ability to detect biological, chemical, and physical contaminants. Another challenge in food safety is simultaneous quantification of viable, dead, VBNC, and biofilm bacterial cells and may be achievable using Raman spectroscopy in the future. Advanced chemometrics [107] and machine learning techniques [108] should be continuously explored so as to improve the analytical power of SERS for food safety surveillance. Overall, Raman spectroscopy has great potential for application in food safety once challenges are addressed and multiplex and quantification capabilities are further improved.

## 7. Conclusions

This review summarizes the recent developments of Raman spectroscopic methods (i.e., Raman spectroscopy, SERS, and Raman microscopy and imaging) for application in food safety. Several studies have demonstrated that Raman spectroscopic methods can identify microbes, toxins, environmental contaminants, additives, adulterants, pesticides, allergens, and plastic particles in foods, which enables their use for the detection of food hazards in the supply chains. Raman spectroscopy has been shown to be a reliable, rapid, specific, and sensitive identification method that can be set up easily and fast and is available as a portable and handheld system. However, several challenges need to be addressed to make Raman spectroscopy a practical detection tool in the food industry. Current hurdles include establishing a universal database of reference spectra, standardization of sample preprocessing, setup of spectrometer and data analysis, screening complex foods in factory- or farm-based settings, and implementation of easy-to-use end-user devices for a variety of analytes. In conclusion, Raman spectroscopic methods are a promising tool to determine biological, chemical, and physical hazards in food systems.
